# Structural basis for chemically-induced homodimerization of a single domain antibody

**DOI:** 10.1038/s41598-019-38752-y

**Published:** 2019-02-12

**Authors:** Jean Lesne, Hung-Ju Chang, Angelique De Visch, Matteo Paloni, Philippe Barthe, Jean-François Guichou, Pauline Mayonove, Alessandro Barducci, Gilles Labesse, Jerome Bonnet, Martin Cohen-Gonsaud

**Affiliations:** Centre de Biochimie Structurale, CNRS UMR5048, INSERM U1054, Université de Montpellier, 29 rue de Navacelles, 34090 Montpellier, France

## Abstract

Chemically-induced dimerization (CID) systems are essential tools to interrogate and control biological systems. AcVHH is a single domain antibody homo-dimerizing upon caffeine binding. AcVHH has a strong potential for clinical applications through caffeine-mediated *in vivo* control of therapeutic gene networks. Here we provide the structural basis for caffeine-induced homo-dimerization of acVHH.

## Introduction

Chemically-induced dimerization (CID) is a ubiquitous mechanism in which two proteins are brought in close proximity by a specific ligand^1^. Several synthetic CID systems have been engineered. The most popular are the FK506 binding protein (FKBP) homodimer and its derivative FKBP/FRB which heterodimerize upon rapamycin binding. Other systems include the GyrB protein and its ligand coumermycin. Researchers have used synthetic CID systems to control protein localization, signalling pathways, split protein activity, or transcription^2^. CIDs were also used to engineer Boolean logic gates operating in living cells^3^.

One promising development for synthetic dimerization systems is their use to control synthetic biological networks activity for clinical applications. For example, the FKBP system was used to control the activity of CAR-T cells or improve their safety^4,5^. In this context, having multiple orthogonal CIDs systems would improve the precision and complexity of therapeutic biological networks.

An alternative to the established CID systems is the anti-caffeine VHH (acVHH), which dimerizes upon caffeine binding with a stoichiometry of two VHH domains for one caffeine molecule^6,7^. We recently used acVHH to control the activation of synthetic bacterial receptors^8^. Importantly, acVHH was used to control glycemia in a diabetes animal model in a caffeine-dependent manner^9^. As such, acVHH has a high potential for therapeutic applications as caffeine is non-toxic, cheap, has no side effects and is not naturally present in the human body. We thus sought to explore the structural basis of ac-VHH/caffeine recognition and ligand-induced homodimerization.

We overexpressed and purified ac-VHH from *E. coli* and obtained crystals only in presence of caffeine (Supplementary Material and Methods). The crystals diffracted at a 2.0 Å resolution. We solved the structure of the complex using molecular replacement (Supplementary Table 1). The asymmetric unit contains 4 VHH dimers (Supplementary Table 1). Monomer and dimer structures are almost perfectly identical with a very low mean RMSD of 0.26–0.4 Å and ~0.6 Å, respectively (Supplementary Fig. 1). Each dimer binds one caffeine molecule buried at the interface.

**Figure 1:**
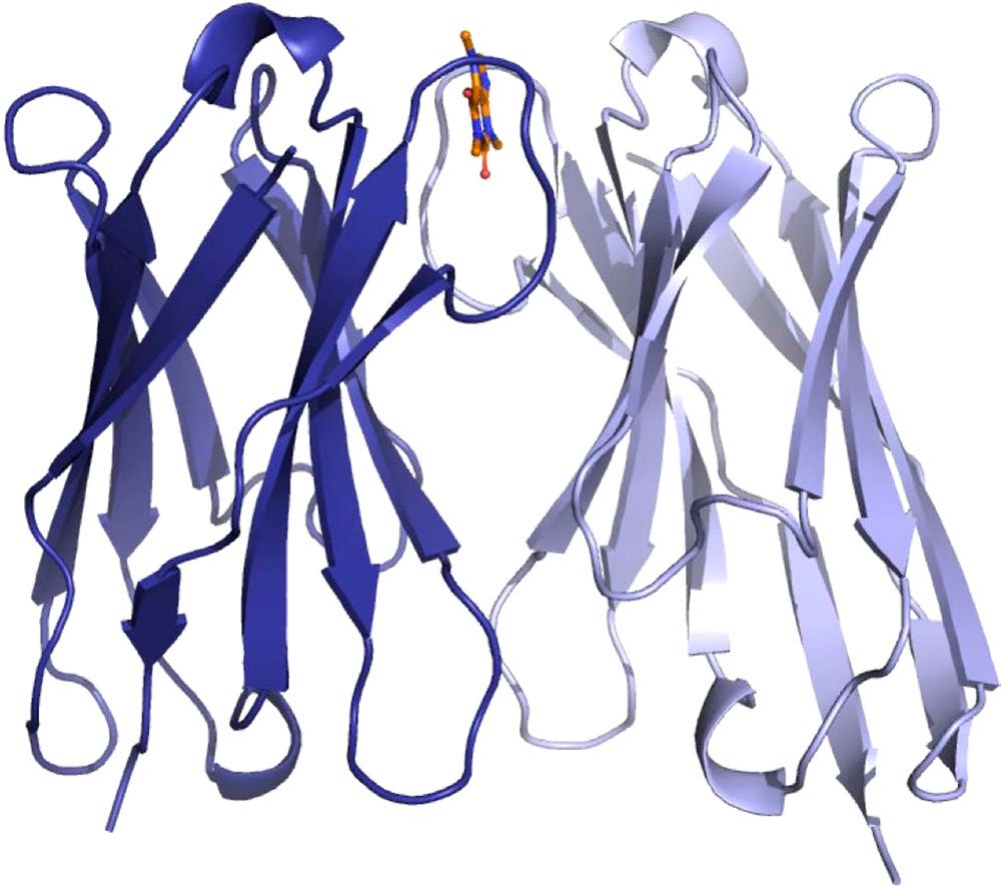
AcVHH dimer in complex with caffeine. Cartoon representation of the acVHH dimer x-ray structure. The caffeine is represented as sticks in orange/blue/red.

**Figure 2:**
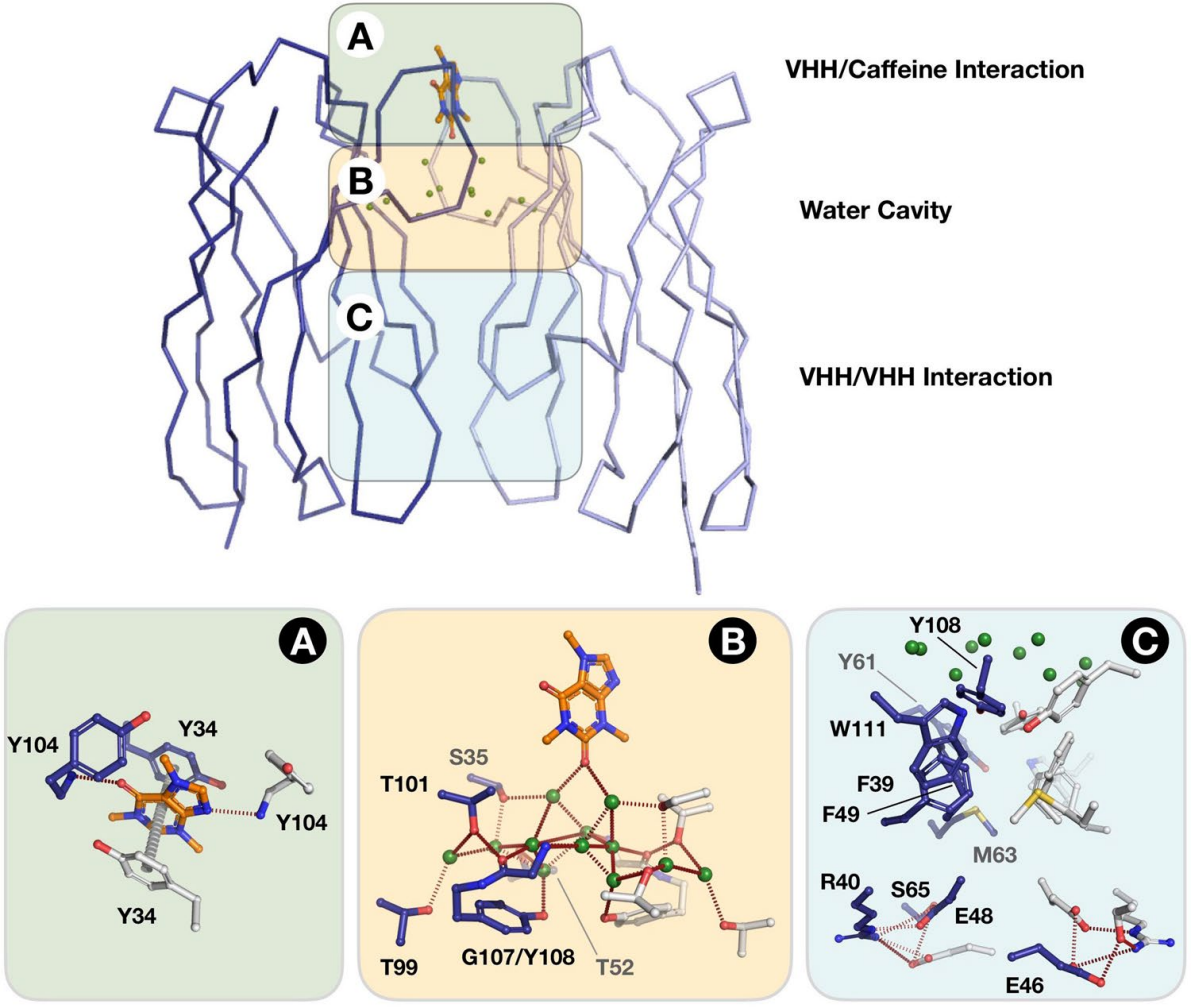
Caffeine/VHH and VHH dimer interface. Detailed of the acVHH/caffeine interaction. The interface is decomposed in three areas and involved the same residues from both acVHH monomers.

The caffeine molecule is stacked on one extremity of the dimer interface (Fig. 1). Only the same two tyrosines, Tyr34 from CDR1 and Tyr104 from CDR3 of each monomer are in direct contact with the ligand through hydrogen bonds and π-π stacking and hydrogen bonds, respectively (Fig. 2). We identified three areas within the dimerization interface: (A) the caffeine/VHH interaction area, (B) a water-filled cavity, (C) the VHH/VHH interaction area. As mentioned, the caffeine/VHH interaction is only limited to few direct interactions (Fig. 2). Two tyrosine, Tyr34 and Tyr34′ (where ‘stands for second monomer’) sandwich the caffeine and form π-π stacking interactions on both sides of the caffeine purine ring. The lateral chains of both tyrosine Tyr104 cap the binding site. The caffeine is an asymmetric molecule, but both main chain NH of Tyr104 and Tyr104′ form an H-bond with the ligand, one with the carbonyl in position 6 of the purine ring and one with the N9 (Fig. 2 – area A). Accordingly, two polar atoms of caffeine form short hydrogen bonds (2.7 and 2.9 Å) to the protein while the third polar group (carbonyl C=O at position C2 on the heterocycle ring) is hydrogen bonded to two symmetrical water molecules. In parallel, the hydrophobic methyl groups make van der Waals contacts to the aromatic ring of Tyr104 (N7-methyl) and the methyl group of threonines Thr101 and Thr101′. These interactions features explain the observed specificity against closely related biomolecules such as theophylline or guanine.

The structure was refined to resolution limit of 2.25 Å, and many water molecules were identified. Among them, 12 were present in all the four independent dimers interface of the asymmetric crystallographic unit (Fig. 2 – area B). Caffeine-induced dimerization engulfs complex network of water molecules that are shielded from the bulk solvent. The ligand is in direct contact with 2 water molecules (see above) and these water molecules are themselves connected with 2 water molecules and the lateral chain of the both Ser35 and Ser35′ (Fig. 2 – area B). These water molecules are in turn interacting with 6 other water molecules in interaction with each other, and with the backbone of Gly107 and with the lateral chain of Ser35 (CDR1), Thr52, Thr99 (CDR3) and Tyr108 (CDR3) from both monomers. The two Tyr108 close the water cavity and are also part of the hydrophobic VHH/VHH interaction surface (Fig. 2 – area C).

The VHH/VHH interaction surface comprises a hydrophobic surface of 850 A^2^ that comprise the residues Phe39, Phe49, Met63, Tyr61, Tyr108 and Trp111 from each monomer. The hydrophobic packing (composed of Phe37, Tyr100B, and Trp103) around the boundaries of the CDR3 is conserved among VHH antibodies and has been described as important for VHH domain stability^10^. In addition, the VHH/VHH interaction surface also comprises residue forming H-bounds and 2 salt-bridges (Arg40, Glu46, Glu48 and Ser65) (Fig. 2 – area C).

**Figure 3:**
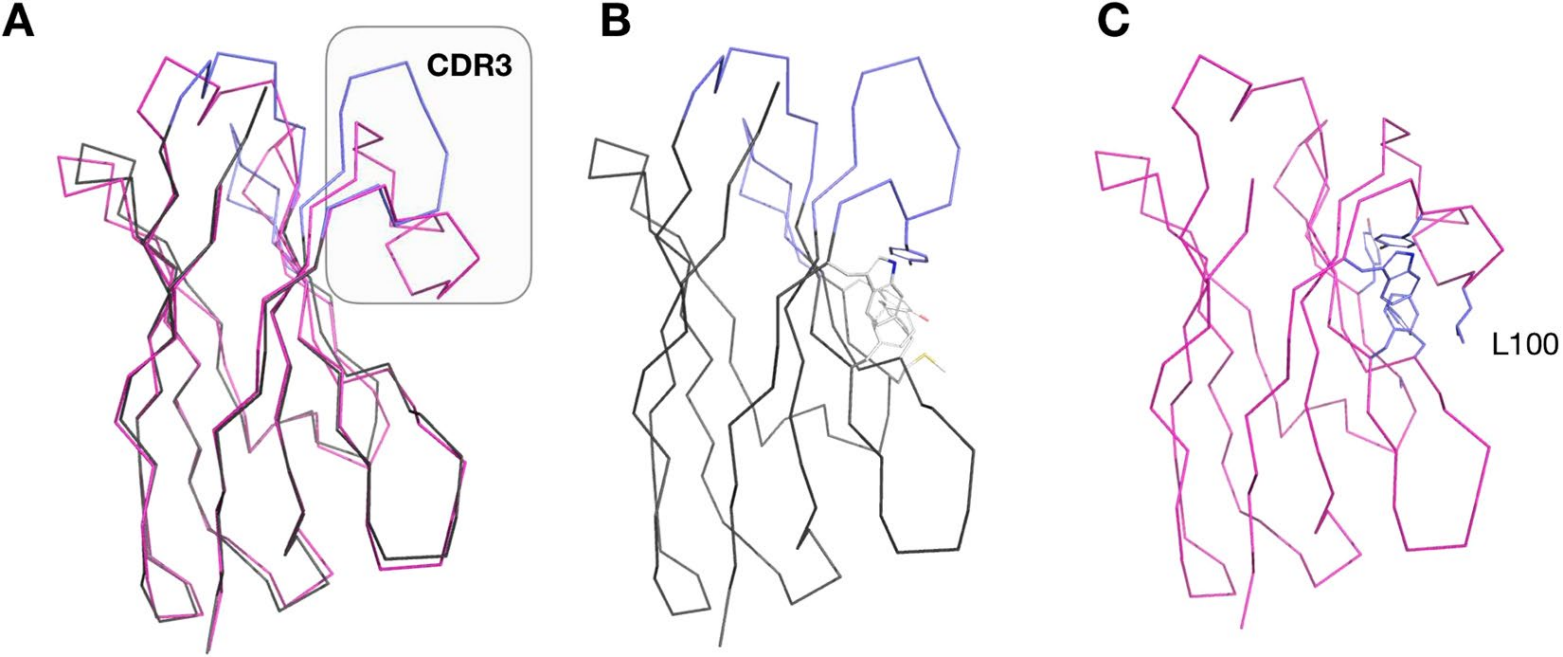
Caffeine/VHH and VHH dimer interface. (**A**) Ribbon representation of acVHH (grey and blue) and the llama nanobody PorM_01 (PDB5LZ0, purple) superimposition. The acVHH CDR loop are represented in blue, the grey box highlights the CDR3 loops. (**B**) The short CDR3 loop of acVHH allows a patch of conserved hydrophobic residues to be exposed and dimerization to happen. (**C**) In “classic” nanobodies, the longer CDR3 loop masks those hydrophobic residues, for the llama nanobody PorM_01 the Leu100 shield the hydrophobic residues.

One remarkable difference between acVHH and conventional VHHs is its short CDR3 loop region. It is composed of only 10 amino acids, which is far shorter than the average 16 amino acid camelid CDR3^11^ (Fig. 3). Therefore, the short CDR3 loop of AcVHH do not to shields this hydrophobic surface like in conventional VHHs^12^. As a consequence, the acVHH residues Phe39, Phe49, Tyr108 and Trp111 become solvent accessible and are able to interact within a dimer (Fig. 2 – area C).

We further investigated the conformational flexibility of the system by performing atomistic, explicit solvent Molecular Dynamics (MD) simulations of the acVHH monomer and of the apo and caffeine-bound homo-dimers. Surprisingly, the caffeine-binding CDR loop is rather rigid in VHH monomer in this timescale and inter-chain interactions and/or the presence of the caffeine do not further hamper the limited conformational dynamics of VHH, with the exception of few tyrosine (Tyr34, Tyr61, Tyr104). For the caffeine-binding homodimer simulations, the most relevant structural fluctuations correspond to a transient yet sizable opening of the VHH/VHH interaction area, which suggest that other regions of the interface, such as caffeine/VHH interaction surface and water molecule cavity, may ultimately be more relevant for stabilizing the overall dimeric architecture.

This study provides a deeper understanding of the structural determinant for caffeine-induced dimerization of acVHH. AcVHH binding mode is different from previously known anti-hapten-VHH structures (*i.e*. PDB1I3U, PDB1QD0, PDB3QXV), in which the hapten molecule binds into a pockets formed by multiple CDR loops belonging to a single VHH^13–15^. For instance, Rr1 dye accommodates in a groove formed by CDR2 and CDR3 of the VHH. In acVHH, caffeine binds into a cavity formed by highly rigid CDR1 and CDR3 from two different VHH molecules, and triggers their dimerization. The binding mode between acVHH and caffeine shares similarities with conventional hapten-antibodies interactions in which haptens bind into a pocket formed by CDRs from both variable heavy and light chain^16^.

A key feature promoting acVHH dimerization is the shorter CDR3 loop. Consequently, hydrophobic residues are exposed and compose most of the VHH/VHH interaction surface. The hydrophobic surface of the acVHH dimer interface is composed by conserved residues involved in the hydrophobic interface between the VH domain and the VL domain in scFV. These residues are also conserved between acVHH and human consensus VHH. On the other hand, the conserved interaction between Pro-L44 and Leu-H45 in antibodies variable domain is replaced by residues Arg40, Glu46, Glu48, and Ser65 which form H-bonds and 2 salt-bridges in acVHH dimer (Fig. 2 – area C). However, this hydrophobic surface neither promotes self-dimerization of the VHH nor changes its stability^7^. The homodimerisation is a direct consequence of the caffeine binding and the set-up of an important and unusual network of interaction between water molecules and the two VHH molecules.

One limitation of current CID systems is their lack of scalability. AcVHH, as a single domain antibody amenable to mutagenesis and selection approaches, could serve as a suitable platform to generate CID systems responding to new ligands. The structure presented here suggests that the large water cavity created by acVHH dimerization and stabilized by caffeine could accommodate other molecules with different size and geometries.

## Supplementary information

SUPPLEMENTARY MATERIALS

## References

[1] Stanton, B. Z., Chory, E. J. & Crabtree, G. R. Chemically induced proximity in biology and medicine. *Science* **359** (2018). 10.1126/science.aao5902 PMC6417506 29590011

[2] DeRose R, Miyamoto T, Inoue T (2013). Manipulating signaling at will: chemically-inducible dimerization (CID) techniques resolve problems in cell biology. Pflugers Arch..

[3] Miyamoto T (2012). Rapid and orthogonal logic gating with a gibberellin-induced dimerization system. Nat. Chem. Biol..

[4] Wu C-Y, Roybal KT, Puchner EM, Onuffer J, Lim WA (2015). Remote control of therapeutic T cells through a small molecule-gated chimeric receptor. Science.

[5] Gargett T, Brown MP (2014). The inducible caspase-9 suicide gene system as a ‘safety switch’ to limit on-target, off-tumor toxicities of chimeric antigen receptor T cells. Front. Pharmacol..

[6] Ladenson RC, Crimmins DL, Landt Y, Ladenson JH (2006). Isolation and characterization of a thermally stable recombinant anti-caffeine heavy-chain antibody fragment. Anal. Chem..

[7] Sonneson GJ, Horn JR (2009). Hapten-induced dimerization of a single-domain VHH camelid antibody. Biochemistry.

[8] Chang H-J (2018). A Modular Receptor Platform To Expand the Sensing Repertoire of Bacteria. ACS Synth. Biol..

[9] Bojar D, Scheller L, Hamri GC-E, Xie M, Fussenegger M (2018). Caffeine-inducible gene switches controlling experimental diabetes. Nat. Commun..

[10] Bond CJ, Marsters JC, Sidhu SS (2003). Contributions of CDR3 to VHH Domain Stability and the Design of Monobody Scaffolds for Naive Antibody Libraries. J. Mol. Biol..

[11] Sircar A, Sanni KA, Shi J, Gray JJ (2011). Analysis and modeling of the variable region of camelid single-domain antibodies. J. Immunol..

[12] Bannas P, Hambach J, Koch-Nolte F (2017). Nanobodies and Nanobody-Based Human Heavy Chain Antibodies As Antitumor Therapeutics. Front. Immunol..

[13] Spinelli S, Tegoni M, Frenken L, van Vliet C, Cambillau C (2001). Lateral recognition of a dye hapten by a llama VHH domain. J. Mol. Biol..

[14] Spinelli S (2000). Camelid heavy-chain variable domains provide efficient combining sites to haptens. Biochemistry.

[15] Fanning SW, Horn JR (2011). An anti-hapten camelid antibody reveals a cryptic binding site with significant energetic contributions from a nonhypervariable loop. Protein Sci..

[16] Dengl S, Sustmann C, Brinkmann U (2016). Engineered hapten-binding antibody derivatives for modulation of pharmacokinetic properties of small molecules and targeted payload delivery. Immunol. Rev..

