# Supplementary material for: Structural basis for chemically-induced homodimerization of a single domain antibody
Source: Sci Rep. 2019 Feb 12;9:1840. doi: 10.1038/s41598-019-38752-y (PMC6372657; doi:10.1038/s41598-019-38752-y)
Supplement: Supplementary file 1 — SUPPLEMENTARY MATERIALS [file 41598_2019_38752_MOESM1_ESM.docx]

**SUPPLEMENTARY INFO:**

**Structural basis for chemically-induced homodimerization of a single domain antibody.**

Jean Lesne^a^ ; Hung-Ju Chang^a^ ; Angelique De Visch ; Matteo Paloni ; Philippe Barthe ; Jean-François Guichou ; Pauline Mayonove ; Alessandro Barducci ; Gilles Labesse ; Jerome Bonnet* ; Martin Cohen-Gonsaud*.

**SUPPLEMENTARY MATERIAL AND METHODS**

*E. coli* BL21(DE3) strains containing pETPhos::*acVHH* (Roumestand et al., 2011) were used to inoculate 1 L of LB medium supplemented with ampicillin (100 μg/ml) and resulting cultures were incubated at 37 °C with shaking until A_600_ reached ~0.5. Then, 1 mM final of isopropyl 1-thio-β-d-galactopyranoside was added and growth was continued for 3 hr at 30 °C. The cells were harvested by centrifugation and the resulting cell pellet was resuspended in buffer A (50 mM Tris-HCl pH 8.5, 150 mM NaCl). Cells were then lysed by sonication and cell debris and insoluble materials were removed by centrifugation. The supernatant was loaded into a Hitrap^TM^ IMAC HP column (Amersham biosciences), equilibrated in buffer A and 4 % of buffer B (buffer A supplemented with 500 mM of imidazole). The column was washed with successive applications of buffer A and 4% of buffer B (approximately 30 ml in total) to remove all the impurities and then buffer B was increased over 20 ml to 100%. Fractions containing the acVHH proteins were identified by SDS-PAGE, then pooled and concentrated using a 5 K cut-off concentrator. The protein loaded to a Superdex 75 26/60 (Amersham biosciences) size exclusion column, equilibrated in buffer 20 mM Tris-HCl pH 8.5, 150 mM NaCl. Again, fractions containing the acVHH protein were identified by SDS-PAGE, then pooled concentrated to 8mg/ml and stored at -20°C until required.

The data sets were collected at the European Synchrotron Radiation Facility in Grenoble, France, on ID30A3 beamline. Crystallization conditions were determined by using hanging-drop-based sparse-matrix screening strategy (*Hampton Research* kits). Protein crystals were grown in ten days, only in presence of caffeine, at room temperature, by mixing 1 *µ*l of the protein solution (with or without 1 mM of caffeine) and 1 *µ*l of crystallization buffer (0.2M MgCl2, NaAc pH 5.5 100mM, PEG 2k 25%), equilibrated over 0.5 ml of the same buffer. Prior to data collection, crystals were harvested with a nylon cryo-loop and immediately flash-cooled in liquid nitrogen. Image processing and data scaling were performed using the programs MOSFLM [1], SCALA [2], and TRUNCATE [3] from the CCP4 program suite [4]. The structures were refined by rounds of rebuilding in Coot and refinement using Refmac [5] of the CCP4 suite. Data collection and refinement statistics for crystal structures are presented in supplementary table 1.

MD simulations of the VHH dimer with and without caffeine have been performed with GROMACS 5.1.2 [6] MD code. The dimer formed by chains A and C from the crystal structure, together with the water molecules in the pocket formed by the two protein chains, has been used as initial structure for the MD simulations. For the simulations of the dimer with caffeine, the caffeine molecule from the crystal structure has been kept. The charge of the system was neutralized with counterions.

Protein molecules were modeled using the Amber14sb [7] forcefield, caffeine was modeled with the Generalized Amber Force Field (GAFF) [8], while the TIP3P [9] force field was used to model water molecules. SETTLE [10] algorithm was used to constraint the bond lengths of water molecules to their equilibrium value, and LINCS [11] algorithm was used for the protein molecules, allowing a timestep of 2 fs. The Particle-Mesh Ewald method [12] was used for the calculation of the electrostatic potential. A cut-off of 1.0 nm was used for the Lennard-Jones interactions. Periodic boundary conditions and a rhombic dodecahedron box with a volume of about 360 nm3 were used.

MD simulations were prepared with the following protocol: 1000 steps of energy minimization with the steepest descent algorithm, followed by 100 ps of NVT and 100 ps of NPT simulations, where the positions of the heavy atoms of the protein were restrained to let the water molecules equilibrate at 300 K and 1 bar. Temperature was controlled with the v-rescale algorithm [13] coupling separately protein and non-protein molecules to avoid the hot solvent/cold solute effect [14]. Pressure was controlled with the Parrinello-Rahman algorithm [15].

Two replicas of the dimer with caffeine and two replicas of the dimer without caffeine have been simulated with different initial velocities, for a total length of 2.5 microseconds. Coordinates of the atoms of protein molecules and caffeine have been saved every 10ps for further analysis. The root mean squared deviation (RMSD) of the position of the atoms of protein molecules (Sup. Fig. S1) has been computed using the rms tool from the GROMACS suite.

**Supplementary References:**

1. Battye, T.G. et al. (2011) IMosflm: a new graphical interface for diffraction-image processing with MOSFLM. Acta Cryst. D67, 271-281.

2. Evans P.R. (2006) Scaling and assessment of data quality, Acta Cryst. D62, 72-82

3. Zwartz, P. (2006) Anomalous signal indicators in protein crystallography. Acta Cryst. D61, 1437

4. Winn M. D. et al. (2011) Overview of the CCP4 suite and current developments. Acta. Cryst. D67, 235-242

5. Vagin, A.A. (2004) REFMAC5 dictionary: organisation of prior chemical knowledge and guidelines for its use. Acta Cryst. 2004 D60: 2284-2295

6. Abraham MJ, et al. (2015) Gromacs: High performance molecular simulations through multi-level parallelism from laptops to supercomputers. SoftwareX 1–2:19–25.

7. Maier JA, et al. (2015) ff14SB: Improving the Accuracy of Protein Side Chain and Backbone Parameters from ff99SB. J Chem Theory Comput 11(8):3696–3713.

8. Wang J, Wolf RM, Caldwell JW, Kollman PA, Case DA (2004) Development and testing of a general Amber force field. J Comput Chem 25(9):1157–1174.

9. Jorgensen WL, Chandrasekhar J, Madura JD, Impey RW, Klein ML (1983) Comparison of simple potential functions for simulating liquid water. J Chem Phys 79(2):926.

10. Miyamoto S, Kollman PA (1992) Settle: An analytical version of the SHAKE and RATTLE algorithm for rigid water models. J Comput Chem 13(8):952–962.

11. Hess B, Bekker H, Berendsen HJC, Fraaije J (1997) LINCS: A linear constraint solver for molecular simulations. J Comput Chem 18(12):1463–1472.

12. Darden T, York D, Pedersen L (1993) Particle Mesh Ewald - An N.Log(N) Method for Ewald Sums in Large Systems. J Chem Phys 98(12):10089–10092.

13. Bussi G, Donadio D, Parrinello M (2007) Canonical sampling through velocity rescaling. J Chem Phys 126(1):014101-1-014101-7.

14. Lingenheil M, Denschlag R, Reichold R, Tavan P (2008) The “Hot-Solvent/Cold-Solute” Problem Revisited. J Chem Theory Comput 4(8):1293–1306.

15. Parrinello M, Rahman A (1981) Polymorphic transitions in single crystals: A new molecular dynamics method. J Appl Phys 52:7182–7190.

**Supplementary Table 1.** Data collection and refinement statistics**.**

| **Wavelength** | 0.9677 |
| --- | --- |
| **Resolution range** | 58.4 - 2.25 (2.33 - 2.25) |
| **Space group** | P 1 21 1 |
| **Unit cell** | 83.26 60.99 87.67 90 93.69 90 |
| **Total reflections** | 353192 (36252) |
| **Unique reflections** | 39917 (3138) |
| **Multiplicity** | 9.0 (9.2) |
| **Completeness (%)** | 95.06 (75.66) |
| **Mean I/sigma(I)** | 13.47 (4.16) |
| **Wilson B-factor** | 19.95 |
| **R-merge** | 0.1097 (0.5386) |
| **R-meas** | 0.1162 (0.5703) |
| **R-pim** | 0.03794 (0.1864) |
| **CC1/2** | 0.999 (0.966) |
| **Reflections used in refinement** | 39883 (3137) |
| **Reflections used for R-free** | 1650 (134) |
| **R-work** | 0.1934 (0.2364) |
| **R-free** | 0.2584 (0.3557) |
| **Number of non-hydrogen atoms** | 7957 |
| **macromolecules** | 7292 |
| **ligands** | 56 |
| **solvent** | 609 |
| **Protein residues** | 948 |
| **RMS(bonds)** | 0.007 |
| **RMS(angles)** | 0.94 |
| **Ramachandran favored (%)** | 96.78 |
| **Ramachandran allowed (%)** | 3.11 |
| **Ramachandran outliers (%)** | 0.11 |
| **Rotamer outliers (%)** | 0.00 |
| **Clashscore** | 9.14 |
| **Average B-factor** | 27.64 |
| **macromolecules** | 27.83 |
| **ligands** | 18.75 |
| **solvent** | 26.15 |
| **Number of TLS groups** | 57 |

Statistics for the highest-resolution shell are shown in parentheses.

**Supplementary Figure:**


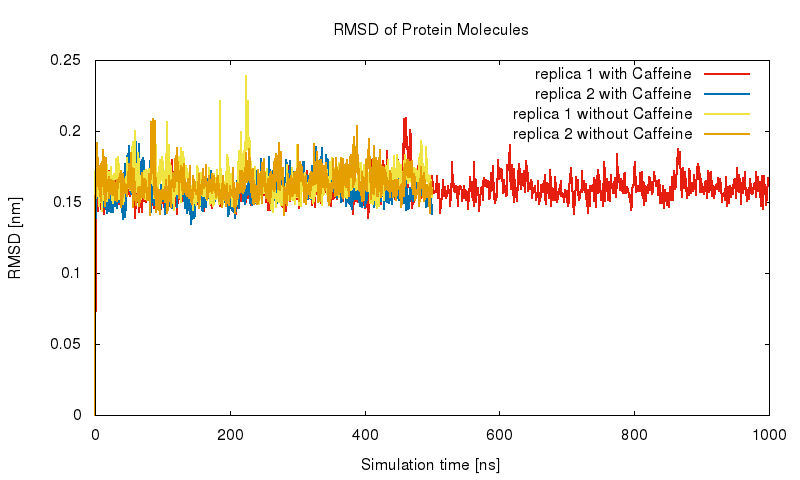


**Sup. Fig. 1:** Root Mean Square Deviation of the protein heavy atoms from the X-ray structure as a function of the simulation time. Curves correspond to indipendent trajectories of the VHH dimer with (red and blue lines) and without caffeine (yellow and orange lines)
